# Evaluation of the Nano-TiO_2_ as a Novel Deswelling Material

**DOI:** 10.3390/molecules21010057

**Published:** 2016-01-04

**Authors:** Ming Chu, Yue-Long Hou, Lan Xu, Zheng-Yun Chu, Ming-Bo Zhang, Yue-Dan Wang

**Affiliations:** 1 Department of Immunology, School of Basic Medical Sciences, Peking University, Beijing 100191, China; hou.yuelong@163.com (Y.-L.H.); xulanyn@163.com (L.X.); 2 Key Laboratory of Medical Immunology, Ministry of Health, Beijing 100191, China; 3 Pharmacy Department, Liao Ning University of Traditional Chinese Medicine, Shenyang 116600, Liao Ning, China; chuzhengyun@163.com (Z.-Y.C.); mbzhang@126.com (M.-B.Z.)

**Keywords:** nano-TiO_2_, nitric oxide, deswelling activity, vascular permeability, nano-TiO_2_ ointment

## Abstract

Nano-TiO_2_ is widely applied in the automobile exhaust hose reels as a catalyst to reduce oxynitride emissions, including nitric oxide (NO). In the biomedicine field, NO plays an important role in vasodilation and edema formation in human bodies. However, the deswelling activity of nano-TiO_2_ has not been reported. Here, we demonstrated that nano-TiO_2_ can significantly degrade the production of NO in LPS-induced RAW264.7 mouse macrophages. Further study indicated that nano-TiO_2_ exhibited an effect on vascular permeability inhibition, and prevented carrageenan-induced footpad edema. Therefore, we prepared a nano-TiO_2_ ointment and observed similar deswelling effects. In conclusion, nano-TiO_2_ might act as a novel deswelling agent related with its degradation of NO, which will aid in our ability to design effective interventions for edema involved diseases.

## 1. Introduction

Nitric oxide is a by-product of combustion of the substances in the air, and it is abundant in automobile engines. Because of the large vehicle population, significant amounts of NO_x_ are emitted to the atmosphere. Attention is given to the catalyst system which simultaneously promotes the reduction of NO. Most reports show that NO can be broke into N_2_ and O_2_ by nano-TiO_2_ as a catalyst with the help of UV light and sensible light [1,2]. In mammals including humans, NO is also an important cellular signaling molecule involved in many physiological and pathological processes. NO, known as an endothelium-derived relaxing factor (EDRF), is a powerful vasodilator with a short half-life of a few seconds in the blood. The endothelium of blood vessels uses NO to signal the surrounding smooth muscle to relax, thus resulting in vasodilation [3,4,5]. Recent studies have shown that NO is also essential for host innate immune responses to pathogens such as viruses, bacteria, fungi, and parasites, which is mainly generated by macrophages [6,7,8,9]. However, excessive production of NO is a common feature of most diseases associated with infection and acute or chronic inflammation, which contributes to edema formation and pain sensitization [10,11].

In the present study, we examined whether nano-TiO_2_ might act as a deswelling material. To gain insights into the molecular mechanism, we further investigated the effects of nano-TiO_2_ on degrading NO and inhibiting vascular permeability. We believe that further study on nano-TiO_2_ will aid in our ability to design effective interventions and treatments for edema involved diseases.

## 2. Results and Discussion

### 2.1. Effects of Nano-TiO_2_ on LPS-Induced NO Production

Nano-TiO_2_ is demonstrated to be active on NO absorption in the air. In mammals including humans, NO is mainly generated by phagocytes (monocytes, macrophages, and neutrophils). Here, we used RAW264.7 mouse macrophages to investigate the effects of nano-TiO_2_ on LPS-induced NO production *in vitro*.

**Figure 1:**
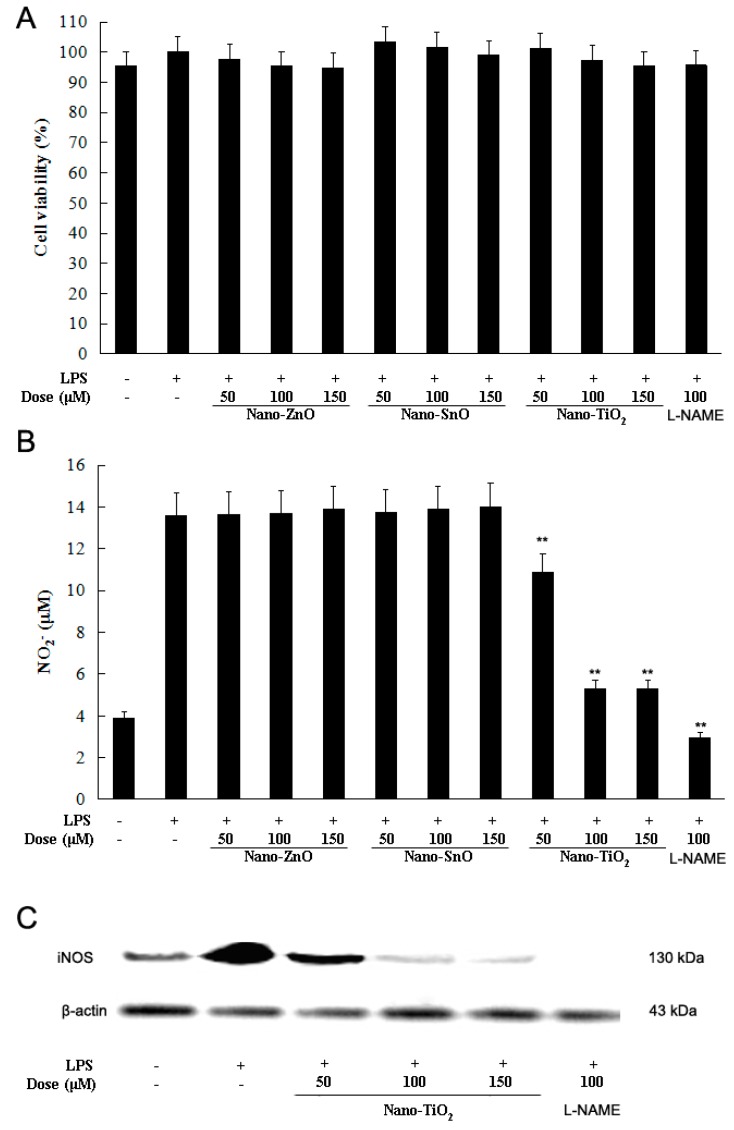
Effects of nano-TiO_2_ on cell viability and nitric oxide (NO) production in LPS-induced RAW 264.7 mouse macrophages. After 24 h treatment, cell viability (**A**) was evaluated by MTT assay and NO production (**B**) was measured by the Griess reaction. Normal group was treated with media only. Control group was treated with LPS (100 ng/mL) alone. Data were shown as means ± standard deviation (SD) of three independent experiments. ** *p* < 0.01 against control group; (**C**) The expression of iNOS was dectected by Western blot.

We first measured the cytotoxicity of nano-materials in RAW264.7 cells by using the MTT assay. RAW264.7 cells were cultured with LPS (100 ng/mL) in the presence or absence of nano-materials. As shown in Figure 1A, nano-ZnO, nano-SnO and nano-TiO_2_ at the concentrations of 50, 100 and 150 μM had no cytotoxic effect.

NO is a relatively unstable molecule, which is produced at low concentrations and rapidly converted into nitrate within 10 s of its formation [12]. Thus, the concentration of nitrate is commonly used to reflect the levels of NO production. Following 24 h of LPS stimulation, a higher level of NO production were measured in the culture media of RAW264.7 cells treated with LPS alone when compared to the untreated cells. However, the significant induction of NO production was reduced by nano-TiO_2_ in a dose-dependent manner, whereas other nano-materials did not reduce the nitrate levels (Figure 1B). We further demonstrated that the expression of iNOS in macrophages decreased after treated with nano-TiO_2_ at the concentration of 50, 100 and 150 μM. Thus, nano-TiO_2_ might be a candidate material for relieving vasodilation concerned with excessive NO production.

**Figure 2:**
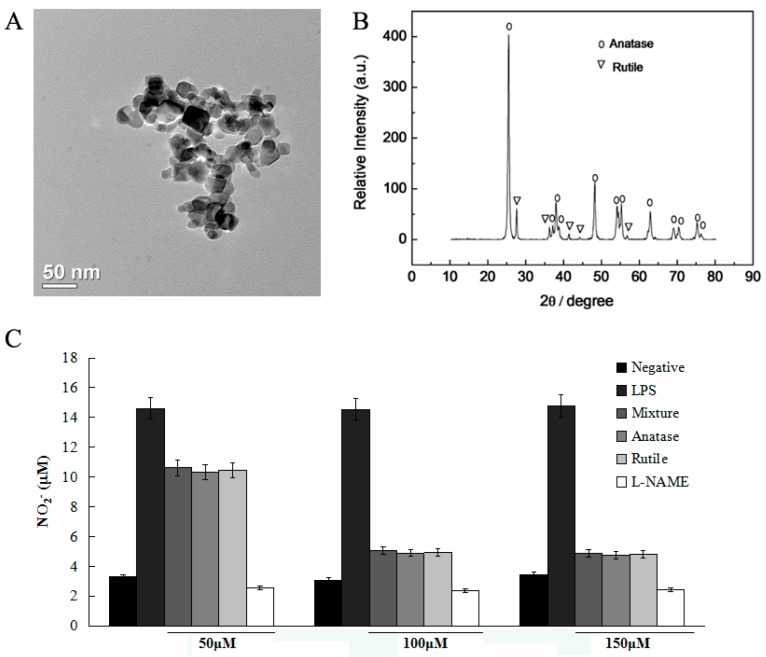
Analysis of nano-TiO_2_. (**A**) Nano-TiO_2_ was measured by electron microscope to a diameter of 21 nm; (**B**) Nano-TiO_2_ was analyzed by XRD method, and separated into anatase and rutile type; (**C**) Anatase and rutile nano-TiO_2_ presented NO degradation action with no significant differences. Data were shown as means ± SD of three independent experiments.

Moreover, we analyzed the nano-size of the nano-TiO_2_ used in this study, and separated it into anatase and rutile type (Figure 2). Then, we detected the effect of nano-TiO_2_ in different types on NO degradation. Both anatase and rutile nano-TiO_2_ presented NO degradation action with no significant differences (Figure 2C). Thus, we chose the anatase nano-TiO_2_ for the further study.

### 2.2. Effects of Nano-TiO_2_ on Inhibiting Vascular Permeability

In 1980, Furchgott and Zawadsko reported the crucial role endothelium in the relaxation of arterial smooth muscle by acetylcholine. The report was based on the integrity of endothelial cells and suggested that the endothelial cells might generate a special transfer molecule causing vascular smooth muscle cell (VSMC) relaxation. Thus, they named this molecule as endothelium-derived relaxing factor (EDRF) [3,4]. Later in 1986, Furchgott and Ignarro further proved that NO is the specific transfer molecule that played the role of EDRF by using spectral analysis of hemoglobin [5]. It is now known that NO can induce the synthesis of cyclic guanosine monophosophate (cGMP) through guanylyl cyclase (GC) leading to relaxation of myosin [13,14,15,16]. Thus, we wondered if nano-TiO_2_ might present an inhibitory effect on vascular permeability via degradation of NO *in vivo*.

**Figure 3:**
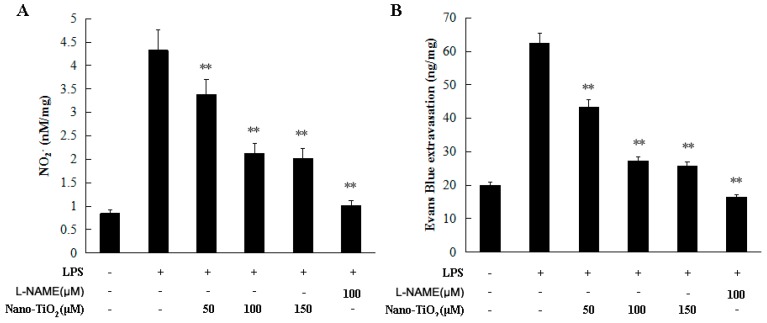
Effects of nano-TiO_2_ on inhibiting vascular permeability. SD rats were injected with 50, 100, and 150 μM nano-TiO_2_ for 1 h prior to the addition of LPS stimulation, compared to 100 μM L-NAME. (**A**) NO_2_^−^ in the skin was detected using the Griess method; (**B**) Evans blue extravasation was used to evaluate the vascular permeability. Data were shown as means ± SD of three independent experiments. ** *p* < 0.01 against control group.

We first investigated the inhibitory effect of nano-TiO_2_ on vascular permeability in female SD rats. The rats were subcutaneous injected with 50 μM, 100 μM and 150 μM nano-TiO_2_ for 1 h prior to the addition of LPS stimulation. NO production decreased significantly in the nano-TiO_2_ treated group (Figure 3A). Evans blue extravasation was employed to evaluate the vascular permeability [17]. As shown in Figure 3B, Evans blue extravasation was significantly decreased in the nano-TiO_2_ treated groups in a dose dependent manner when compared to the rats stimulated with LPS alone (*p* < 0.01).

**Figure 4:**
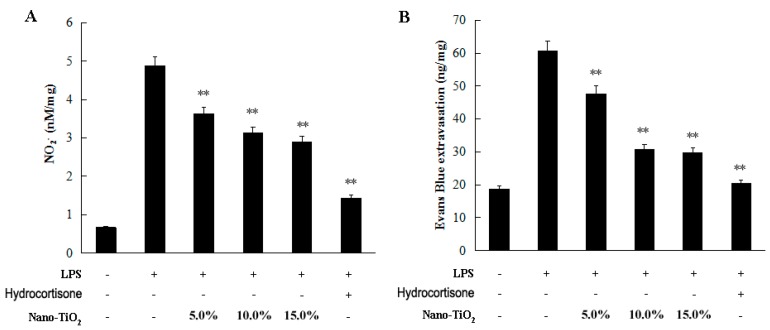
Effects of nano-TiO_2_ ointment on inhibiting vascular permeability. Ointment contained 5.0%, 10.0%, and 15.0% of nano-TiO_2_ were rubbed on the depilated dorsal skin of rats. (**A**) NO_2_^−^ in the skin was detected using the Griess method; (**B**) Evans blue dye extracted from the skin was measured. Data were shown as means ± SD of three independent experiments. ** *p* < 0.01 against control group.

Nano-TiO_2_ is a white pigment widely used in foods, sunscreens, and cosmetic products [18,19,20,21]. Here, we prepared ointments which contained 5.0%, 10.0% and 15.0% of nano-TiO_2_ with vaseline and lanolin in the ratio of 1:2 as an accessory. Three points were marked on the median line of the depilated dorsal skin, where 2 mg of the nano-TiO_2_ ointment was rubbed carefully for 1 min on a circular area with a 2 cm diameter with each point. The nano-TiO_2_ ointment was applied 1 h before LPS subcutaneous injection. NO production decreased significantly in the nano-TiO_2_ treated group (Figure 4A). More importantly, the vascular permeability was significantly inhibited by nano-TiO_2_ ointment in a dose dependent manner when compared to the rats treated with accessories (Figure 4B).

### 2.3. Effects of Nano-TiO_2_ on Carrageenan-Induced Paw Edema

An increase in vessel wall permeability contributes to the formation of edemas. Here, we used the carrageenan-induced paw edema model to evaluate the deswelling effect of nano-TiO_2_ [22,23,24].

**Figure 5:**
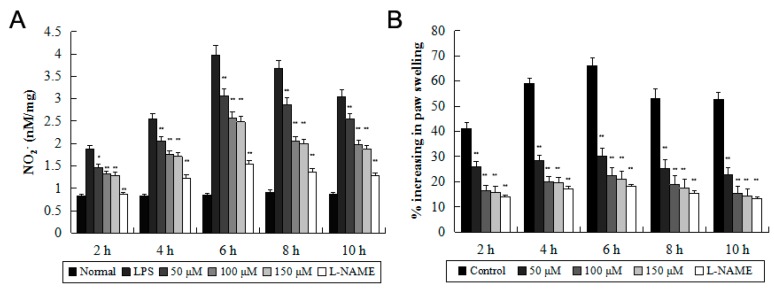
Effect of nano-TiO_2_ on carrageenan-induced paw edema. 0.1 mL of nano-TiO_2_ (50–100 μM) was administered 1 h before carrageenan stimulation. (**A**) NO_2_^−^ in the footpad was detected using the Griess method; (**B**) The degree of swelling in the footpads was measured using a plethysmometer. Data were shown as means ± SD of three independent experiments. * *p* < 0.05 against control group; ** *p* < 0.01 against control group.

In this study, SD rats were divided into five groups (eight animals in each group), and 0.1 mL of nano-TiO_2_ was administered 1 h before carrageenan stimulation. NO production decreased significantly in the footpad of nano-TiO_2_ treated rats (Figure 5A). In addition, subcutaneous injection of nano-TiO_2_ resulted in a significant reduction in rat paw edema. The deswelling effect of nano-TiO_2_ at doses of 50–150 μM was statistically significant for reducing paw edema of rats at 2, 4, 6, 8 and 10 h after induction of edema (Figure 5B).

**Figure 6:**
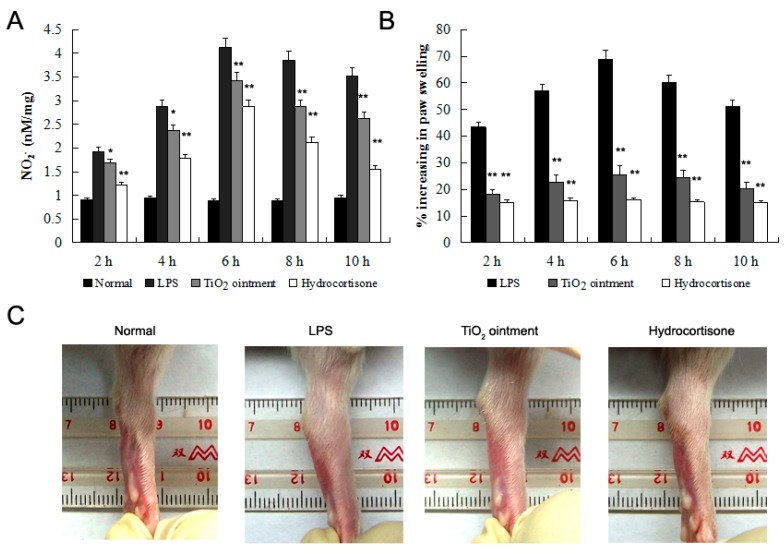
Effect of nano-TiO_2_ ointment on carrageenan-induced paw edema. Ointment contained 10.0% of nano-TiO_2_ was smeared on the SD rats’ right footpads 1 h prior to the addition of carrageenan induction. (**A**) NO_2_^−^ in the footpad was detected using the Griess method; (**B**) The degree of swelling in the footpads was measured using a plethysmometer; (**C**) The degree of swelling in the foot pads was photographed. Data were shown as means ± SD of three independent experiments. * *p* < 0.05 against control group; ** *p* < 0.01 against control group.

Then, we evaluate the inhibitory effect of nano-TiO_2_ ointment on carrageenan-induced paw edema. The volumes of the unilateral hind paws of these animals were measured, and on each paw, nano-TiO_2_ ointment was carefully rubbed 1 h before the carrageenan was given. This ointment-treated part was covered softly with a fiber cloth in order to prevent the rats from licking off the ointment. Then, carrageenan was injected subcutaneously into the paw, and the volume of the hind paw was measured at 2, 4, 6, 8, and 10 h. Ointment with 10% nano-TiO_2_ showed a significant reduction of NO release and rat paw edema (Figure 6). Carrageenan-induced edema develops through mediators in three phases. The early phase is caused by histamine release, the second phase is mediated by kinin, and the late phase is caused by prostaglandins. According to the NO degradation effects of nano-TiO_2_, we proposed that nano-TiO_2_ are effective for the first phase of edema formation.

**Figure 7:**
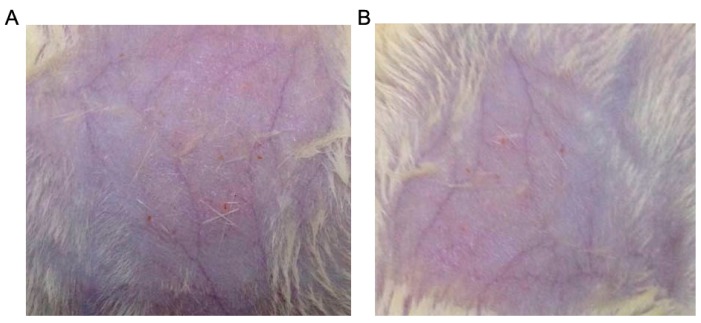
Irritation of nano-TiO_2_ ointment on the skin of rats. Accessory (**A**) and ointment with 15% nano-TiO_2_ (**B**) was rubbed on the skin of rats for month.

Moreover, the application of the ointment with 15% nano-TiO_2_ to the skin of the rats for 1 month had no irritation on the animal skin (Figure 7). It is reported that nano TiO_2_ does not appear to significantly penetrate the intact skin [25]. However, the compromised skin allows nano-sized particle penetration through the skin [26]. Therefore, we believe that the nano-TiO_2_ ointment is available for the prevention and treatment of edema formation in the skin wounds.

## 3. Experimental Section

### 3.1. Animals

All experiments was performed in compliance with relevant laws and institutional guidelines and approved by the Peking University Biomedical Ethics Committee. Female SD rats (4–6 weeks old) were provided by Beijing Laboratory Animal Research Center. The experimental animals were housed separately at room temperature (20 ± 2 °C), humidity 55%–60%.

### 3.2. Cell Culture

RAW264.7 mouse macrophages were purchased from American Type Culture Collection (ATCC, Manassas, VA, USA) and cultured in Dulbecco’s modified Eagle’s medium (DMEM, Gobico, NY, USA) supplemented with 100 µg/mL of penicillin/streptomycin and 10% heat-inactivated fetal bovine serum (FBS, Gibco, NY, USA), in a humidified atmosphere of 5% CO_2_ at 37 °C, until reaching 80% confluency [27]. The medium was changed every 3 days.

### 3.3. Nano Materials

Nano-TiO_2_, nano-ZnO and nano-SnO were purchased from Shanghai Huijing Sub-Nanoseale New Material Co., Ltd. (Shanghai, China). To prepare nano TiO_2_ ointment, we mixed vaseline and lanolin in the ratio of 1:2 as an accessory and then mixed with nano-TiO_2_ in different proportions.

### 3.4. Griess Method

We used a total Nitrate/Nitrite Parameter Assay Kit for determining NO according to the instructions (Catalog KGE001, R & D, Minneapolis, MN, USA) [28]. The completed reaction was read at 540 nm. The concentrations of NO_2_^−^ in the cell culture supernatant were expressed as NO_2_^−^ (µM). In addition, the concentration of NO_2_^−^ in the skin or the footpad of rats were calculated as NO_2_^−^ divided by the weight of tissue (nM/mg).

### 3.5. Western Blot Analysis

Total protein of RAW-264.7 cells were extracted and quantified respectively. After SDS-PAGE, the protein was transferred to polyvinylidene fluoride membrane. The membrane was incubated with antibodies against iNOS and β-actin (Sigma-Aldrich, Saint Louis, MO, USA) overnight at 4 °C. Then the membrane was incubated with the secondary antibodies and detected with enhanced chemiluminescence kit.

### 3.6. Vascular Permeability Assay

Evans blue dye at a concentration of 2% (40 mg/kg; Sigma-Aldrich) was injected into the great saphenous vein of 4- to 6-week-old mice. After 60 min, 2 cm-diameter free back skin graft was removed, blotted dry, and weighed. The Evans blue dye was extracted from the skin with 1 mL of formamide overnight at 55 °C and measured spectrophotometrically at 630 nm [17].

### 3.7. Carrageenan-Induced Paw Edema Method

The deswelling effect of nano-TiO_2_ were evaluated by the carrageenan-induced paw edema method [22]. Nano-TiO_2_ was administered 1 h before the rats received 0.1 mL of carrageenan (1%, *w*/*v*) into the subplantar area of the right hind paw. The control group was treated with the vehicle only. The paw volume of rats was measured before injecting carrageenan and at 2, 4, 6, 8, 10 h after carrageenan stimulation using a plethysmometer (MK-101P, Tokyo, Japan). The edema was expressed as the increase in paw volume, and the percentage of inhibition of edema was expressed as the reduction in volume with respect to the control group [24].

### 3.8. Statistical Analysis

Data are expressed as means ± SD of three replicate determinations and were analyzed by SPSS (SPSS Inc., Chicago, IL, USA) [29]. Statistical significance was determined by one way Analysis of Variance (ANOVA). Data were regarded as statistically significant when *p* < 0.01.

## 4. Conclusions

At the onset of an infection, macrophages undergo activation and release NO responsible for vasodilation. Increased permeability of the blood vessels results in an exudation of plasma proteins and fluid into the tissue, which manifests itself as swelling. During the edema formation process, nano-TiO_2_ plays an important role in reducing NO generated by phagocytes *in vivo*, so as to inhibit vascular permeability, and reduce swelling. In addition, we further prepared nano-TiO_2_ ointment, and proved it to be effective on deswelling. These results suggest that nano-TiO_2_ might act as an deswelling material through reducing NO, which will aid in our ability to design effective interventions and treatments for edema involved diseases.
